# Report on Webinar Series Cell and Gene Therapy: From Concept to Clinical Use

**DOI:** 10.3390/pharmaceutics14010168

**Published:** 2022-01-11

**Authors:** Christopher F. van der Walle, Christine Dufès, Arpan S. Desai, Julie Kerby, Joanne Broadhead, Alice Tam, Zahra Rattray

**Affiliations:** 1 GlaxoSmithKline, R&D, Gunnels Wood, Stevenage SG1 2NY, UK; chris.vanderwalle@ct.catapult.org.uk; 2 Strathclyde Institute of Pharmacy and Biomedical Sciences, University of Strathclyde, Glasgow G4 0RE, UK; c.dufes@strath.ac.uk; 3 Advanced Drug Delivery, Pharmaceutical Science, R&D, AstraZeneca, Cambridge CB2 0RE, UK; Arpan.Desai@astrazeneca.com; 4 Manufacturing, Cell and Gene Therapy Catapult, Stevenage SG1 2FX, UK; julie.kerby@ct.catapult.org.uk; 5 Freeline, Stevenage SG1 2FP, UK; Joanne.Broadhead@freeline.life; 6 Royal Marsden Hospital (NHS), London SW3 6JJ, UK; Alice.Tam@rmh.nhs.uk; 7 Academy of Pharmaceutical Sciences, c/o Bionow, Greenheys Business Centre, Manchester Science Park, Pencroft Way, Manchester M15 6JJ, UK

**Keywords:** cell therapy, gene therapy, similars, manufacture, in vivo gene therapy, viral vectors

## Abstract

With the launch of the UK Academy of Pharmaceutical Sciences Advanced Therapy Medicinal Products Focus Group in late 2020, a webinar series reviewing the current and emerging trends in cell and gene therapy was held virtually in May 2021. This webinar series was timely given the recent withdrawal of the United Kingdom from the European Union and the global COVID-19 pandemic impacting all sectors of the pharmaceutical sciences research landscape globally and in the UK. Delegates from the academic, industry, regulatory and NHS sectors attended the session where challenges and opportunities in the development and clinical implementation of cell and gene therapies were discussed. Globally, the cell and gene therapy market has reached a value of 4.3 billion dollars in 2020, having increased at a compound annual growth rate of 25.5% since 2015. This webinar series captured all the major developments in this rapidly evolving area and highlighted emerging concepts warranting cross-sector efforts from across the community in the future.

## 1. Proceedings

The Academy of Pharmaceutical Sciences (APS- the UK Pharmaceutical sciences community) hosted a virtual webinar series in May 2021 on the topic of cell and gene therapy, holistically combining concepts from bench discovery to their clinical implementation. This was a flagship event for the Advanced Therapy Medicinal Products (ATMP) Focus Group which was launched in fall 2020. The session was attended by delegates representing the academic, pharmaceutical industry, pharmacy practice and regulatory sectors; a diversity which was reflected in the speaker line-up and panel discussion. The global ATMP market is a rapidly growing sector, reaching a market worth of 7.9 billion dollars in 2020 [1]. As of October 2021, there are >20 cell and gene therapy products that are licensed from the FDA Office of Tissues and Advanced Therapies (OTAT) [2]. In 2020, 154 ATMP clinical trials were documented in the UK alone, representing a 12% global share of ongoing ATMP trials and evidence of UK leadership in this area [3]. The majority of ATMPs under investigation in the UK are based on viral vectors, with the portfolio of therapies under investigation showing equal representation of both ex vivo and in vivo gene therapies.

Ex vivo strategies for genetic alteration include cell therapies with a rapidly growing global market predicted to reach 45.4 billion dollars by 2028 [4]. Cell therapies may be broadly referred to as autologous cell therapies in which direct isolation of cells from patients, their culture, expansion and genetic modification takes place outside the body. This is followed by infusion of the genetically altered autologous cells to elicit a therapeutic response. Allogenic cell therapies exemplified by mesenchymal stem cells or pluripotent stem cells can be manufactured at a commercial scale and are available as off the shelf products [5].

**Figure 1:**
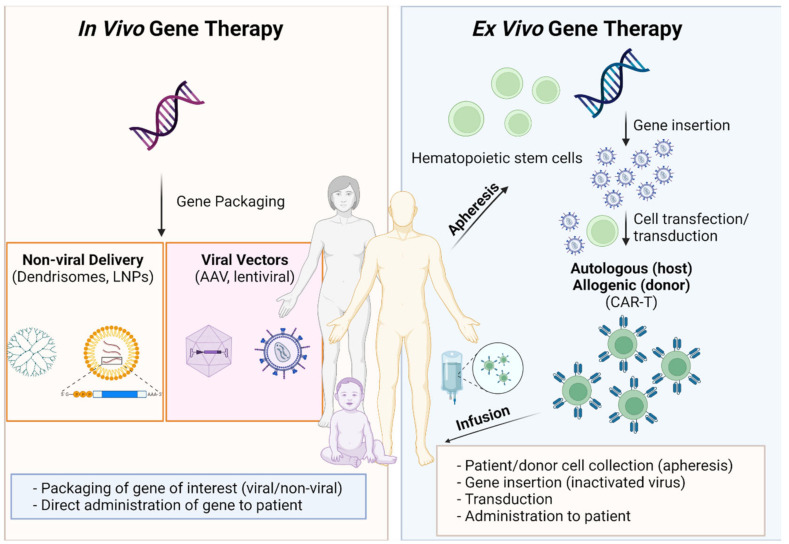
A summary of gene therapy technologies available that were discussed at the Cell and Gene Therapy symposium, created with Biorender.com, accessed on 15 October 2021.

In vivo ATMPs include the use of alternative gene delivery systems that may be viral (e.g., adeno-associated virus or lentiviral) or non-viral (e.g., polymeric and lipid nanoparticles) in composition. Targeting of specific tissues is required for the successful delivery of genetic materials with in vivo therapies [6]. Since the FDA-approval of the first siRNA-based drug product patisiran in 2018, two further products (givosiran 2019 and lumasiran 2020) have been approved and a further seven are under phase III clinical trial investigation. Recent rapid advancements in drug delivery technologies and characterization of their quality attributes have led to the design and engineering of non-viral delivery systems for the delivery of genetic materials (Figure 1).

Chris van der Walle (GSK, Director, Formulation Cell & Gene Therapy) opened the presentations with a talk entitled ‘Formulation considerations for T-Cell Therapies’. Dr van der Walle presented an outline of the autologous chimeric antigen receptor (CAR) T cell therapy landscape. The challenges to CAR/TCR T-cell treatment of solid tumours arise from their heterogeneous antigen expression, poor infiltration, immunosuppressive microenvironment and limited persistence [7]. While these challenges are addressed in next-generation dual CARs, increased cell numbers will be required for each dose unit, placing a demand for greater cell expansion during manufacture. The recently approved lisocabtagene maraleucel (Breyanzi^®^, Juno/Bristol Myers Squibb (New York, NY, USA), 2021) [8] broke conventions of drug product fill into infusion bags seen with other CAR-T cell therapy products, through adopting vials (5 mL, cyclic olefin copolymer). The manufacture of drug products for infusion is currently complex and costly, and starts with the requirement to generate a recombinant viral vector (filed as wither a ‘drug substance’ or ‘critical starting material’) able to transduce the T cells with the target transgene. Transient transfection of adherent HEK293T cells with four plasmids (the target transgene, viral envelope glycoprotein, gag-pol, and rev) facilitates the generation of lentiviral material under GMP for clinical supply. The complexity of this process can be reduced by using a suspension cell line stably transfected with a single large bacterial artificial chromosome DNA construct encoding all vector components [9]. Lentiviral vector harvest and cell clarification, nucleic acid degradation and purification processes continue to evolve [10]. Significant losses of lentiviral vector may also be experienced during the sterile-filtration step (i.e., 0.2 µm filtration); the positioning of this step depends on the overall process design and may be eliminated through the design of an entirely closed aseptic process [11]. It also remains necessary to find a suitable formulation buffers and primary containers for fill, since lentiviral vectors are prone to degradation following frozen storage (−80 °C) and subsequent thaw (freeze–thaw) at the point of T cell transduction.

Central to the presentation was the concept of a ‘vein-to-vein time’ that, for the autologous T cell process, begins with apheresis of a patient’s peripheral blood mononuclear cells and ends with infusion of the DP. It was noted that the cost of goods (COGs) for cell and gene therapies is a topic of hot debate, wherein the Chemistry, Manufacturing and Controls (CMC) of T cell therapies contributes almost 50% to the external project expenditure (costs minus staffing) and over 40% of internal project expenditure. The major contributor is the so-called ‘patient COGS’, followed by process development for vector and T cell, including their technology transfer to a CMO, plasmids and cell banks (Table 1).

With the focus of the presentation being formulation, specific references were made to cryopreservation agents and freezing. The former universally remains dimethyl sulfoxide (DMSO) for T cells entering the clinic and the latter generally involves the use of a liquid nitrogen-controlled rate freezer, prior to long term storage in vapour phase liquid nitrogen (−150 °C) or liquid nitrogen (−196 °C).

With regard to the lentiviral vector starting material, its fill-finish into either cryogenic vials or bags likely depends on the compatibility of these primary containers with the intended expansion system for the T cell drug substance, e.g., a closed process for T cell expansion may demand aseptic welding of a bag containing the lentiviral vector material.

The T cell formulation must maintain a drug product that is practically free of visible particulates prior to freezing and storage. Promising methods are currently under development for reporting subvisible particulates that may one day find application pending their qualification [13] With the move towards closed, aseptic processes both for vector critical starting material and cell drug product, there is a need to design and characterise custom single-use systems. An understanding of the properties of single-use system components and procedures for aseptic sealing and welding of plastic tubing are critical to maintaining drug product sterility. The presentation concluded with questions and answers, and it was noted that manufacture of T cell drug product under GMP is well established at several clinical sites worldwide [14].

Arpan Desai (AstraZeneca Pharmaceuticals, Team leader gene therapies) presented on advances in the biological understanding of lipid nanoparticle delivery for mRNA therapeutics. He discussed the biological barriers to the systemic delivery of non-viral drug delivery systems [15,16]. Subsequently Dr Desai discussed the key attributes and considerations for CRISPR-Cas based gene editing systems, considerations for the design and development of lipid nanoparticle (LNPs) systems as mRNA delivery systems, their cellular internalization and intracellular fate [17]. He presented an imaging-based machine learning phenotypic screen for the assessment of functional gene delivery using fluorescence-based (e.g., m-Cherry and eGFP) reporter systems in a diverse panel of cancer cell lines derived from various tissue origins. This machine learning tool known as ‘Advanced Cellular and Endocytic Profiling for Intracellular Delivery’, ACE-ID has been used to evaluate functional gene delivery. Findings from this work has revealed rapid trafficking of LNPs is one of the hallmarks of high transfection cell lines. Detailed assessment of functional delivery of LNPs was performed in the H358 cell line known for a high rate of endosomal entrapment [18]. Targets that were found to contribute to dramatic improvements in functional gene delivery included phosphatidylinositol at the plasma membrane, early endosomes and the cytoskeleton. Such phenotypic screens can enable high throughput initial evaluation of prototypes for mechanistic characterization during early discovery stages [16,18]. The talk by Dr Desai concluded with the design of a modified LNP formulation process which could evade the clathrin-mediated endocytosis and utilize micropinocytosis for cellular internalization [15]. This talk concluded that machine learning tools can be implemented in the early interrogation of LNP prototypes and identify key biology that can be exploited for efficient drug delivery. This strategy can be implemented for the rational design of LNP formulations with desired target product profiles to evade biological barriers routinely encountered by gene delivery systems.

Christine Dufes (Reader in Nanomedicine, Strathclyde Institute of Pharmacy and Biomedical Sciences) discussed challenges in the design of novel gene therapies in oncology drug development. Her presentation focused on the implementation of dendrimers and dendrimer-based vesicles (dendrisomes) [19,20,21] with endogenous ligand surface-functionalization (i.e., lactoferrin and transferrin) to increase gene delivery and subsequent efficacy in difficult to treat tumours. Her presentation included a discussion of in vitro and early pre-clinical findings following administration of novel targeting prototypes [22] in prostate [23] and skin cancer models. Findings reported in this presentation included a significant increase in cellular internalization relative to non-functionalized delivery systems and significant tumour regression [24,25].

The second webinar session included a presentation on the UK manufacture landscape for cell and gene therapies and was followed by an interactive panel discussion with the presenters from across all three webinar sessions covering the manufacture considerations for the rapidly growing complex medicines landscape.

Julie Kerby (Cell and Gene Therapy Catapult, Director of commercialization) delivered a presentation introducing the CGT and its remit in the UK cell and gene therapy manufacture landscape. The CGT was set up in 2012 by the UK government and is a part of a network of catapults with the remit to bridge the academic and industry sector translational gap by embedding technologies and innovations in the UK ATMP landscape. The UK is largest CGT cluster outside the US with 26 manufacturing facilities supporting early to large-scale manufacture of ATMPs and >90 innovator companies. The UK hosts 12% of all global ATMP clinical trials. She discussed the industry challenges of developing complex cell and gene therapies using autologous cell therapies and gene therapies as examples. Some of these were attributed to variable starting materials in terms of the genetic variability, clinical treatment history and the supply chain elements of developing these therapies. There is a fast turnaround time involved required and novel analytical methods are required for rapid release tests to accelerate the timelines involved in their development and release to patients.

She then discussed the challenges commonly encountered with gene therapy based technologies were described as supply chain issues associated with the fill-finish steps. The variability in the viral gene therapy analytical space was cited as a driver for increasing the cost and size of batch. The scale-up costs for early clinical campaigns drives the costs associated with developing novel gene therapies. The CGT supports the analytical and manufacturing challenges encountered by companies and academia to accelerate the commercial translation and technology-readiness of these products. During the pandemic, the CGT has been actively involved in the delivery of the COVID-19 vaccine. Dr Kerby then presented the partners and facility sites (Braintree, Stevenage) for the CGT in the UK, discussing the non-competitive space that has contributed to key developments in tackling challenges with fill-finish steps. Her presentation concluded with futureproofing expansion of the CGT industry, where she referred to a survey conducted by the CGT of the UK ATMP workforce studying staff numbers and skilled workforce requirements.

**Figure 2:**
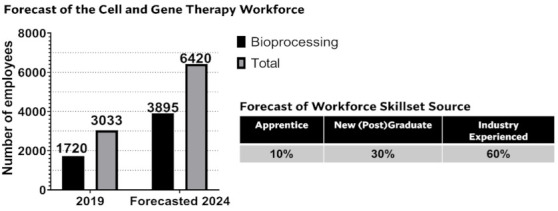
Findings from the CGT Catapult survey of the UK ATMP workforce, including the workforce statistics with a forecasted 112% increase in the total workforce and 126% increase in bioprocessing roles within the cell and gene therapy industry (left). Forecast of the proportion of contribution from various skillset sources to the cell and gene therapy industry (right), adapted from [26].

In response to the survey, the CGT launched an apprenticeship scheme with >10 programmes developed across a range of technical skill areas and competencies which was the first apprentice programme of its own type. This scheme has led to over 130 apprentices being recruited and employed into the CGT industry sector [26] (Figure 2).

The final webinar session in the series covered Chemistry, Manufacturing and Control (CMC) and regulatory considerations for the development of in vivo gene therapies and the implementation of ATMPs in the clinic setting.

Adeno-associated virus (AAV) vectors have now been investigated in >140 clinical trials with over ten years follow-up data available on their safety and efficacy. Dr Joanne Broadhead (VP, CMC project delivery at Freeline Therapeutics) discussed regulatory and CMC challenges encountered in the development of recombinant AAV gene therapy vectors from an analytical and formulation perspective. She provided a market overview of in vivo gene therapies, describing the key Zolgensma^®^ and Luxturna^®^ drug product features as commercially available drug products. Dr Broadhead then discussed the challenges in analytical method development for in vivo gene therapies, citing the heterogeneity of packaged DNA and the potential for fragment annealing and extension that may contribute to efficacy. She then presented some of the typical critical quality attributes (CQAs) for AAV drug products and key analytical considerations at the DNA, capsid and drug product levels Figure 1 [27]. The influence of drug product CQAs on AAV product quality, safety and efficacy is not always apparent, warranting further investigation of the in vivo impact of gene therapy CQAs.

Viral vector delivery systems have historically been formulated in surfactant-containing (poloxamer, polysorbate) buffers and stored at low temperatures (i.e., −80 °C). The cost of goods associated with developing viral gene therapies has limited the range of formulation studies and investigation of more sophisticated formulation options (e.g., lyophilization).

**Figure 3:**
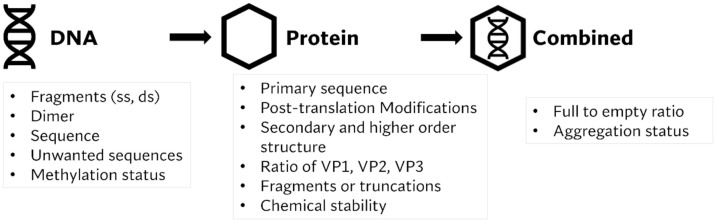
Product Critical Quality Attributes for viral in vivo gene delivery systems at DNA, protein and combined levels. ss: single strand, ds: double strand, vp: viral particles.

Dr Broadhead then discussed the challenges associated with drug substance (DS) manufacture, which include process complexity, reliance on multiple starting materials, multiple processing steps and control of process parameters are known to influence product CQAs (Figure 3). A deeper understanding of process impact on yield and DNA packaging is required to drive the successful manufacture of in vivo gene therapies. The current regulatory landscape for viral gene delivery systems is ever-evolving with expectations from manufacturers changing over time. She then discussed considerations for in vivo gene therapy drug product (DP) manufacture, which are typically a standard biologics fill/finish process with sterile filtration prior to aseptic filling. Batch sizes for in vivo gene therapies are typically small, leading to high costs per dose; therefore, processes are designed to minimize product wastage. Sampling for release and stability testing of gene therapies are generally based on low-volume methods and reuse of sample from non-destructive techniques.

Reference samples for quality assurance should be of sufficient size to allow the performance of at least two full analytical measurements on the batch though the feasibility of this due to scarcity of material in small scale production has been acknowledged by regulatory bodies. In line with sample consumption requirements, container closure integrity (CCI) testing of vials containing buffer has been accepted by regulators as sufficient for clinical trial materials [28].

Dr Broadhead then discussed the regulatory challenges and opportunities in the development of in vivo gene therapies. One of the key regulatory hurdles was the ever-evolving regulatory landscape for ATMPs as a deeper product understanding is being developed over time. However, with this evolution platform approaches can be leveraged to deliver various genes of interest across multiple areas of clinical need. Data from existing products can be used to support the development of new products for other disease targets or clinical indications, provided that the same platform is used (e.g., capsid manufacturing process and testing regime). The FDA and EMA have issued guidance documents on the CMC considerations for human gene therapy investigational new drug applications [29] and quality, non-clinical and clinical aspects of gene therapy [30] and advanced therapy medicinal products in clinical trials [28].

Some of the current hot topics in AAV manufacture were cited as empty/full ratio, where the number of empty capsids is quantified, with regulators raising questions around the impact of empty capsids on safety and efficacy. Many manufacture processes are currently under development to remove empty capsids from the DS during manufacture. Dosing assays to ensure accuracy and reproducibility of the product dosage are key to ensure efficacy. Digital droplet PCR is increasingly being used, which does not require the use of a standard. There is also increasing scrutiny of in vitro potency assays as the most meaningful indicator of in vivo efficacy.

Alice Tam (Lead Pharmacist for ATMPs at the Royal Marsden) discussed the clinical application of cell and gene therapies (CGT) within the hospital setting. She presented the distribution of CGTs across therapeutic areas, with over 35% of all CGTs under clinical trial investigation being deployed in the oncology category [3]. She highlighted the rapid pace and expansion of CGT implementation in the clinic as a challenge, and cited the infrastructure and governance requirements within the clinical setting as a key hurdle in ATMP clinical use. To deliver ATMPs safely and effectively, hospitals are required to have sufficient governance structures in place to support the implementation of CGTs as medicinal products, which falls under the remit of hospital pharmacies. However, the complexity of ATMPs and their handling in a clinical setting necessitates input from a multidisciplinary team beyond the hospital pharmacy. The Pan UK Pharmacy Working Group for ATMPs established in 2018, consists of pharmacists with specialism in the administration, prescribing, monitoring and governance of ATMPs. This group has provided a range of literature and guidance documents on the governance and preparation requirements that underpin the clinical application of ATMPs.

In addition to governance requirements for the implementation of ATMPs in the clinical setting, infrastructure requirements include aseptic facilities, appropriate cold-chain storage, and due to the acute toxicities of ATMPs an ITU facility is required onsite for some ATMPs. In addition to drug product handling infrastructure, clinical staff involved in the handling of ATMPs are required to receive training on the complex handling requirements for ATMPs and the management of unique acute toxicity (cytokine release syndrome- CRS and immune effector cell-associated neurotoxicity- ICANS) in patients that often results from ATMP treatment [31]. CRS is the most common toxicity observed following CAR-T cell infusion [32]. Various efforts to date have attempted to classify the severity of CRS, with a four-stage classification being used by the American Society for Transplantation and Cellular Therapy (ASTCT) based on common presenting symptoms (i.e., fever, myalgia, arthralgia) [33]. The onset of ICANS in patients receiving cell therapies can occur from a few days post-administration up to a few weeks. To keep up with the rapidly growing ATMP landscape, it is critical that the clinical workforce are trained in line with the developments within the sector.

**Figure 4:**
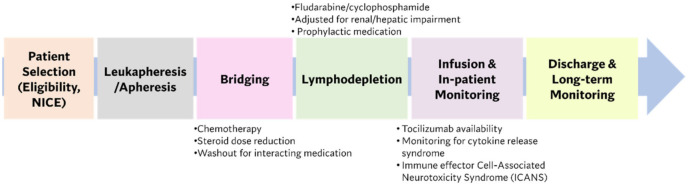
The CAR-T patient journey and various clinical considerations. NICE: National Institute for Health and Care Excellence.

She continued the presentation with a clinical scenario of a CAR-T patient journey and stages involved in CAR-T therapy (Figure 4).

The talk was concluded with a discussion on additional governance structures in terms of organizational policies and requirements, quality systems, and local procedures within the healthcare system.

## 2. Conclusions

The number of innovator ATMPs under development and clinical investigation is rapidly increasing, creating new opportunities for the management of unmet clinical need. The presentations and panel discussions at the Academy of Pharmaceutical Sciences Advanced Therapy Medicinal Products (ATMP) Focus Group symposium provided an insight into the latest developments within the landscape. These included the challenges and opportunities in the development of new cell and gene-based therapies. With the rapid expansion of ATMP research, development and clinical applications there is a clear need for a multidisciplinary and multisector approach in which clinicians interact with scientists in academia, the pharmaceutical industry and regulatory bodies. In the UK, a number of government organizations such as the Cell and Gene Therapy Catapult and professional bodies including the Royal Pharmaceutical Society and Academy of Pharmaceutical Sciences have initiated a portfolio of activities to support the futureproofing of the UK workforce in meeting the demands associated with ATMP implementation and introducing key infrastructure that will support the rapid development and scale-up of emerging innovator products.

## Figures and Tables

**Table 1:** Breakdown of external project expenditure from a commitment to pivotal clinical trial to launch, adapted from [12].

Category	Cost ($M)	Patient COGs Subcategory	% Contribution to Patient COGs
Patient COGs	55	Apheresis	1
Cell, PD & TT *	18	Processing apheresis material	10
Vector, PD & TT	25	GMP vector/batch	26
Plasmids/cell banks	8	GMP T cell process/batch	42
R&D consumables	4	Release and stability testing, per specifications (analytics)	17
Supply chain	4	Qualified Person, release DP	3
		Shipping	2

* Process Development and Tech Transfer.

## Data Availability

Not Applicable.
